# Local Adaptation Shapes Phenotypic and Genetic Diversity in *Zygophyllum loczyi*

**DOI:** 10.3390/genes16070729

**Published:** 2025-06-23

**Authors:** Jan-Cheng Wang, De-Yan Wu, Xue-Rong Li, Jia-Yi Lu, Suo-Min Wang, Qing Ma, Hai-Shuang Liu, Xi-Yong Wang, Jing-Dian Liu, Dao-Yuan Zhang

**Affiliations:** 1 State Key Laboratory of Desert and Oasis Ecology, Key Laboratory of Ecological Safety and Sustainable Development in Arid Lands, Xinjiang Institute of Ecology and Geography, Chinese Academy of Sciences, Urumqi 830011, Chinawangxy@ms.xjb.ac.cn (X.-Y.W.); ariiiiiink@gmail.com (J.-D.L.); zhangdy@ms.xjb.ac.cn (D.-Y.Z.); 2 Xinjiang Key Laboratory of Conservation and Utilization of Plant Gene Resources, Xinjiang Institute of Ecology and Geography, Chinese Academy of Sciences, Urumqi 830011, China; 3 Turpan Eremophytes Botanical Garden, Chinese Academy of Sciences, Turpan 838008, China; 4 Institute of Economic Forest, Xinjiang Academy of Forestry, Urumqi 830092, China; 5 College of Forestry and Landscape Architecture, Xinjiang Agricultural University, Urumqi 830052, China; 6 State Key Laboratory of Herbage Improvement and Grassland Agro-Ecosystems, College of Pastoral Agriculture Science and Technology, Lanzhou University, Lanzhou 730020, China

**Keywords:** phenotypic traits, differentiation, habitat, desert population, evolutionary strategy

## Abstract

**Background/Objectives:** Desert plants exhibit remarkable resilience to extreme environments, and their capacity for population establishment is noteworthy. However, the adaptation process mechanisms of those plants to harsh habitats, particularly concerning intraspecific differentiation and genetic diversity, remain poorly understood, and a comprehensive framework is lacking. *Zygophyllum loczyi* Kanitz, an annual or biennial desert herb, demonstrates significant phenotypic plasticity across diverse habitats. **Methods:** Using mixed-effects models, this study examined 20 populations from four deserts to assess phenotypic variation and predict trait_environment relationships. **Results:** The findings indicated substantial inter-population phenotypic differentiation in *Z. loczyi*, with greater variation observed between deserts than within them. Traits such as blade length, petal length, sepal length, and stamen length were influenced by environmental conditions. Mixed-effects model prediction showed that the growth location of *Z. loczyi* significantly impacted its phenotypic traits. The characteristics of the four desert populations displayed varying responses to temperature and moisture changes, with the most pronounced response noted in the Gurbantunggut desert (Gt) population, indicating that survival stress has an important influence on the performance of plants. The single nucleotide polymorphisms result further confirmed that the differentiation and genetic diversity of the Gt population displayed the highest selection pressure, resulting the small effective size of the population. **Conclusions:** This study uncovers the adaptive mechanism of *Z. loczyi* to habitat through investigating the inter-population phenotypic differentiation and genetic diversity and provides new insight into local adaptation and evolutionary processes in the desert environment.

## 1. Introduction

Phenotypes represent the morphological and structural traits of organisms, shaped by the interplay of genetic expression and long-term environmental adaptation [1,2]. Phenotypic variation plays a pivotal role in species evolution and ecological adaptation [3]. Phenotypic diversity focuses on the phenotypic variation of plant populations in heterogeneous habitats. Local adaptation occurs when populations evolve divergent adaptive traits in response to spatially heterogeneous selection pressures [4]. Plant phenotypes arise from genotype environment interactions over evolutionary timescales and serve as observable manifestations of adaptive evolution [5]. After long-term natural selection and environmental stress, plants usually have phenotypic variation, which is also a survival strategy for plants to adapt to different habitats [6,7]. Phenotypic diversity serves as the foundation for germplasm resource characterization and utilization, representing a critical component in contemporary conservation studies while providing a direct and efficient approach to assess genetic diversity [8].

Phenotypic diversity represents the observable expression of plant genetic diversity, with geographical clines reflecting evolutionary patterns of morphological adaptation to environmental gradients [9]. As the fundamental level of biodiversity, genetic diversity reflects the richness of a species’ genes and determines a species’ adaptive potential to environmental change [10]. Plant genetic diversity is shaped by endogenous factors, such as breeding systems, genetic drift, selection, mutation, and gene flow, and exogenous pressures from environmental fluctuations and anthropogenic disturbances [11]. Local adaptation emerges from the interplay of multiple evolutionary factors, including environmental variability, linkage disequilibrium, population history and natural selection [12,13]. Phenotypic analysis serves as a primary methodology for assessing genetic diversity, providing the most tractable proxy for species diversity quantification [14]. Molecular markers bridge this relationship, enabling precise genotype–phenotype linkage analyses [15,16]. Deciphering the mechanisms underlying phenotypic variation and its environmental responsiveness offers critical insights into plant phylogenetic relationships and adaptive evolution.

Deserts cover approximately one-third of the Earth’s terrestrial surface, creating regions characterized by water scarcity and precipitation variability [17]. These extreme environments support specialized vegetation communities, including xerophytes, psammophytes, halophytes, and ephemeral plants. Nevertheless, the evolutionary history of phenotypic traits of desert plants and their molecular adaptation mechanisms remains understudied [18,19]. The increasing frequency of extreme climatic events under global change scenarios aggravates the deterioration of the ecological environment [20,21]. In this case, studies on phenotypic differentiation and genetic molecular mechanisms of desert plants should be carried out to provide a new perspective for understanding species evolution in special habitats [22,23]. The Central Asian deserts (Taklimakan, Gurbantunggut, Badain Jaran, and Qaidam) exhibit pronounced aridity due to their mid-latitude Eurasian positioning. Their fragmented habitats and geographic isolation create ideal natural laboratories for studying population divergence and ecological speciation [24,25].

Plant trait coordination depends upon the studied environmental gradient [26]. With the decrease of temperature, precipitation, and relative humidity of the air, the leaf area, length, petiole length, and leaf aspect ratio of plants decrease significantly [27]. Plant populations inhabiting more xeric habitats exhibit smaller growth rates and higher investment in traits enhancing water uptake (e.g., higher root-to-shoot ratios), and reducing water loss (small sclerophyllous leaves) [28,29]. *Z. loczyi* (Zygophyllaceae) is a rare annual/biennial desert pioneer herb species in this genus. It is widely distributed in a variety of habitats in arid and semi-arid areas in northwest China, has important ecological values such as wind prevention and sand fixation, and plays an important role in maintaining the fragile ecological environment in arid areas [30]. Many species exhibit population divergence due to local adaptation. Across a species’ range, distinct populations may undergo adaptive evolution at varying rates or trajectories [31]. Based on the previous investigation, there are significant differences in phenotypic traits like height, leaves, and fruit size among different populations of the *Z. loczyi* [32], making it an ideal material for studying the local adaptation mechanism of desert plant populations. The necessary experimental materials and molecular biological methods, combined with statistical research methods, can be obtained from field investigations to identify the population’s phenotypic and genetic diversity and understand the genetic variation of the population and its response to the environment. Investigating *Z. loczyi* phenotypic diversity in the four deserts allows us to achieve the critical aims of this paper, which are as follows: (1) assessing phenotypic variation and associated environmental adaptation strategies and (2) assessing genetic diversity underlying desert plant differentiation. *Z. loczyi* is an ideal material for studying the mechanism of population differentiation of desert plants and will help to reveal the basic mechanism and law of differentiation and local adaptation of desert plants and provide scientific support for desertification control and the identification of plant stress resistance genes.

## 2. Materials and Methods

### 2.1. Field Sampling Design

*Z. Loczyi* has an upright stem with many branches at the base. The petiole is shorter than that of the leaflets, which are elliptical or obovate. The peduncle is 2–6 mm long. The petals are nearly ovate, and the stamens are shorter than the petals. The fruit is a cylindrical capsule. It exhibits the key characteristics that make it an ideal model species for studying population diversity in desert plants: (1) a short life cycle (consistent with the generation time hypothesis, which posits that recombination drives mutation accumulation), (2) a broad geographic distribution, (3) occupancy of diverse habitat types, and (4) pronounced phenotypic differentiation correlated with environmental variation.

**Figure 1:**
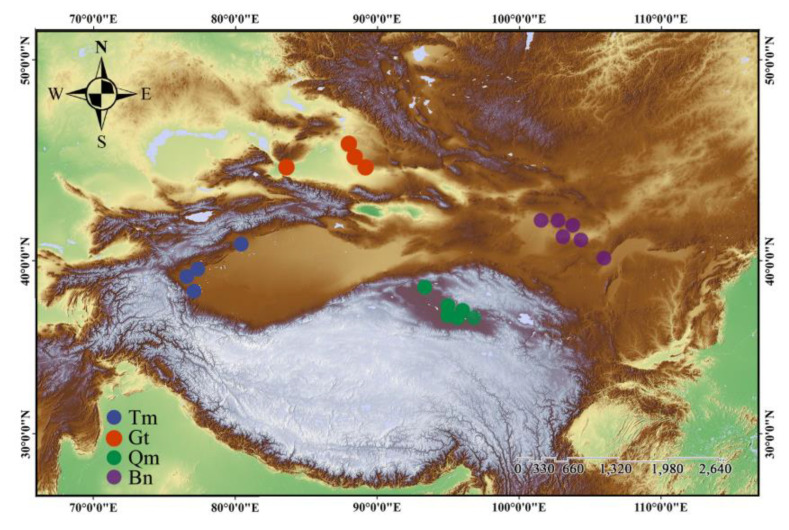
Submap of sampling sites of *Z. loczyi* populations.

Prior to field sampling, the distribution range of *Z. loczyi* in China was determined using data from the China Vegetation Map, the China Digital Herbarium, and records from Xinjiang ecological and geographical studies. From 2020 to 2022, we collected samples from four deserts in northwest China: the Taklimakan Desert (Tm, 64 individuals from 4 populations), the Gurbantunggut Desert (Gt, 64 individuals from 4 populations), the Qaidam Desert (Qm, 99 individuals from 6 populations), and the Badain Jaran Desert (Bn, 104 individuals from 6 populations). In total, 331 individuals from 20 populations were sampled (Figure 1 and Table S1). To minimize the risk of clonal effects from proximity, a minimum distance of 10 m was maintained between any two sampled individuals, and populations were spaced at least 10 km apart to reduce spatial autocorrelation.

### 2.2. Phenotypic Trait Measurements

From the samples collected in the field, we randomly measured the phenotypic traits of individuals and evaluated at least 20 individuals for each population. We measured 12 phenotypic traits [7,29,33], including vegetative growth traits (plant height, stem diameter, blade length/width) and reproductive traits (peduncle length, sepal length/width, petal length/width, stamen length, and fruit length/width), using a digital vernier caliper with a precision of 0.01 mm. Fresh plant samples were lyophilized (freeze-dried) and stored at −80 °C for subsequent molecular analyses.

### 2.3. Environmental Data Collection

Based on field surveys and historical literature, we selected 11 habitat variables and 23 climate variables (Table S2). These variables included climatic parameters (mean, extreme, and seasonal values of precipitation, temperature, wind speed, and solar radiation), topographic factors (altitude, slope, and aspect), and soil properties (soil type and pH). Climate variables were obtained from the WorldClim database (www.worldclim.org). Topographic variables (elevation, slope, and aspect) were derived from 30 m resolution DEM data downloaded from the Geospatial Data Cloud Platform of the Computer Network Information Center, Chinese Academy of Sciences. River distances were calculated as Euclidean distances based on 1:1 million national river data from the same platform. Geographic coordinates (latitude/longitude) were recorded in the field using a GPS receiver during sampling.

### 2.4. SNP Genotyping and Sequencing

We randomly selected 169 individuals (≥6 per population) from the 20 sampled populations. Total genomic DNA was extracted from leaf tissues using the Cetyl Tri-methyl Ammonium Bromide (CTAB) method [34]. The DNA quality and concentration were assessed using 1% agarose gel electrophoresis and a NanoDrop 2000 Spectrophotometer (Thermo Fisher Scientific, Waltham, MA, USA). DNA libraries were prepared by Baimaike Biotechnology Co. (Beijing, China) for whole-genome resequencing. DNA was sheared via ultrasonication to 300–500 bp fragments. DNA ends were repaired using T4 DNA polymerase and Klenow fragment, followed by A-tailing for adapter ligation. Fragments were then amplified via bridge PCR on flow cells. To ensure the accuracy of the detection results, redundant reads were filtered using samtools (v1.9) based on the alignment of cleaned reads to the reference genome. Library quality was verified by agarose gel electrophoresis and quantified before sequencing on the HiSeq6000 platform (PE150 mode) with 10× coverage per sample. Raw reads were filtered to obtain clean data by removing reads with >50% bases having Q-score <10 or >10% N-content. Reads were aligned to the *Zygophyllum xanthoxylum* reference genome [35]. Alignment files were processed using *samtools* (v1.9) to calculate sequencing depth and genome coverage [36].

Single-nucleotide polymorphisms (SNPs) and insertions/deletions (InDels) were identified using *GATK* (v3.8). To ensure detection accuracy, we first filtered redundant reads from the reference-aligned clean sequences using *samtools* (v1.9). Variant calling was performed with the *GATK* HaplotypeCaller algorithm, followed by strict filtering to generate a final set of high-confidence variants in VCF format [37]. To ensure high quality data, we utilized *samtools* to perform strict filtering of SNP calls based on the following criteria: (1) a minimum SNP support number (coverage depth) of more than 3, (2) a minimum allele frequency of more than 0.05, and (3) a deletion rate of less than 10%. Variant identification, programmed to detect SNPs, was conducted with the Unified Genotyper application of the Genome Analysis Toolkit [38].

### 2.5. Data Processing and Statistical Analysis

Phenotypic traits are presented as mean ± standard error (SE). Data normality and homoscedasticity were assessed using Shapiro–Wilk and Bartlett’s tests, respectively. For normally distributed data with homogeneous variance, one-way ANOVA was employed to compare phenotypic differences among the four desert populations. Phenotypic differentiation coefficients within and between deserts were evaluated using two-tailed *t*-tests. Environmental variables were standardized prior to principal component analysis (PCA) using the *prcomp* package to identify key environmental indicators [39]. Redundancy analysis (RDA) was subsequently performed with the vegan package to quantify environment-phenotype relationships. Significant predictors from RDA were incorporated into mixed-effects models using lme4, with environmental factors as fixed effects, sampling sites as random effects, and phenotypic traits as response variables. Models with intraclass correlation coefficients (ICC) > 0.1 were retained for interpretation. All analyses were conducted in R v4.4.1.

To reconstruct the historical divergence timeline, we applied the Sequentially Markovian Coalescent (SMC) approach [40], calibrating the molecular clock using the mutation rate of Oryza sativa [41]. For gene flow detection, we employed *TreeMix* to infer migration directions between populations. The ABBA/BABA method was used to infer introgression events among populations based on asymmetric phylogenetic relationships across four deserts. By analyzing the tree topology and associated statistical outputs from *TreeMix*, to infer patterns of gene flow between populations. The maximum-likelihood population tree topology and residual covariance matrices were analyzed to statistically validate gene flow events.

## 3. Results

### 3.1. Analysis of Phenotypic Differences Among Desert Populations

**Figure 2:**
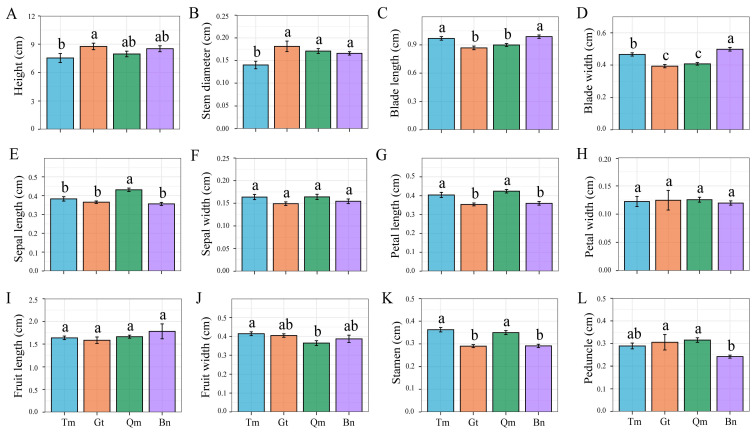
Comparison of phenotypic traits of the *Z. loczyi* in four deserts. Different letters indicate significant difference in four deserts, while the same letters indicate no significant difference. (**A**) Height, (**B**) Stem diameter, (**C**) Blade length, (**D**) Blade width, (**E**) Sepal length, (**F**) Sepal width, (**G**) Petal length, (**H**) Petal width, (**I**) Fruit length, (**J**) Fruit width, (**K**) Stamen, (**L**) Peduncle.

One-way ANOVA revealed significant differences (*p* < 0.05) in most measured traits across the four desert populations, excluding petal width, sepal width, and fruit length (Figure 2). Notably, we observed that Tm has minimal values for plant height and stem diameter, the Gt and Qm populations have reduced leaf dimensions (width), and the Qm population has the largest floral structures (petal length) but the smallest fruits. Trait correlation analysis demonstrated strong associations among floral traits (Figure S1). These results indicated adaptive phenotypic differentiation across desert environments.

Among the 20 populations from the four deserts, the mean coefficient of variation (CV) was 4.07% for populations within the same desert and 6.71% for populations from different deserts. As a whole, phenotypic variation showed that populations in different deserts were more varied than populations in the same desert (Table 1), and the coefficient of variation was small. Notably, we observed significant differences in stem diameter, blade length/width, sepal length, petal length, and stamen length among desert populations. Fruit traits exhibited relatively low variation, suggesting they are evolutionarily conservative, whereas leaf traits showed high variation, indicating phenotypic plasticity. These patterns collectively indicate microenvironmental adaptation within deserts and a continuous differentiation of phenotypic traits across desert ecosystems.

### 3.2. Screening of Environmental Factors and Phenotypic Traits

**Figure 3:**
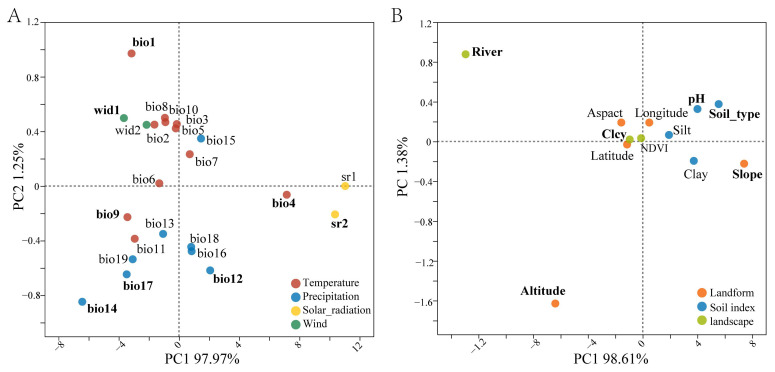
PCA of (**A**) climatic variables and (**B**) habitat variables of *Z. loczyi* populations. Dots and codes correspond to the PCA loadings and names of the climatic variables and habitat variables considered (see Tables S3 and S4). The climatic variables and habitat variables selected for the phenotypic traits analysis are shown in bold text.

Principal component analysis (PCA) of meteorological and habitat variables revealed distinct environmental gradients across the four desert ecosystems (Figure 3). The analysis identified key factors structuring phenotypic variation: meteorological factors (bio1, bio4, bio9, bio12, bio14, bio17, wid1, and srad7) and habitat factors (river, altitude, slope, soil_type, pH, and clcy).

**Figure 4:**
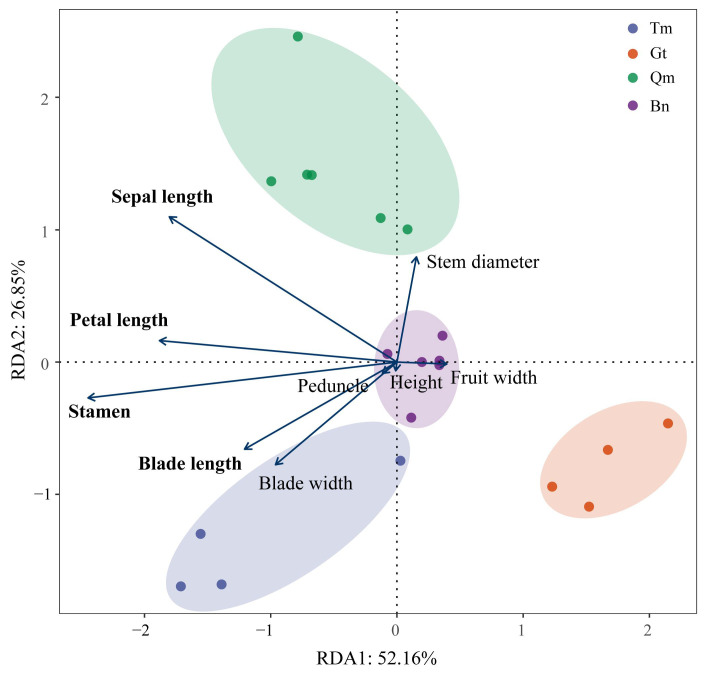
RDA of phenotypic traits and environmental factors.

Redundancy analysis (RDA) based on PCA-selected environmental drivers revealed significant environment–phenotype associations (Figure 4). The first two RDA axes collectively explained 79.01% of constrained variation (RDA1: 52.16%; RDA2: 26.85%), with four traits showing particularly strong environmental responses: stamen length, petal length, sepal length, and blade length. In addition, the environmental factors of 20 populations of the four deserts were clustered in different quadrants, indicating that the environmental factors of the four deserts were relatively different.

### 3.3. Modeling Environment–Phenotype Relationships

**Figure 5:**
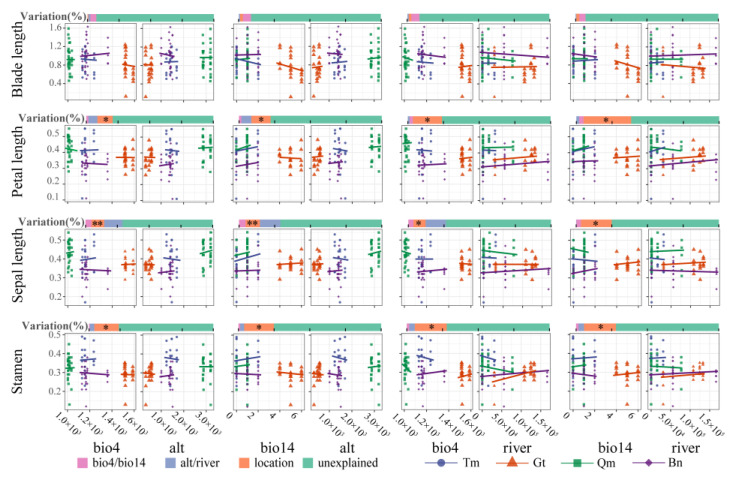
Prediction of phenotypic traits of *Z. loczyi* by mixed effects model. *, *p* < 0.05; **, *p* < 0.01.

A mixed-effect model was used to evaluate the variation of phenotypic traits in response to environmental factors in different desert populations. As shown in the variance decomposition results (Figure 5), environmental factors and location together explained about 25% of the variation in flower traits, among which sampling sites had a significant impact on flower traits. The model explained less than 10% of the variation in blade traits. These results indicated that the flower traits of *Z. loczyi* were influenced by different deserts, while the blade traits were influenced by multiple meteorological and habitat variables.

Under the influence of temperature, precipitation, and altitude, the phenotypic traits of Gt were most affected by the variation of water and heat conditions, and the leaf and flower traits decreased with the increase of the variation (Figure 5). The phenotypic traits of Qm were least affected, and the flower traits were increased by the water change in the driest season. Under the influence of temperature, water, and river, leaf traits decreased, flower traits Gt and Bn increased, and Qm decreased. The fixed effect also had different effects on the performance of *Z. loczyi* in different deserts (Figure S2). The results of the mixed-effects models showed that the phenotypic traits of the four deserts were affected by the environment, and the leaf and flower traits had different responses to temperature and water changes, among which the phenotypic traits of Gt had the strongest influence.

### 3.4. Genetic Diversity Analysis of Different Desert Populations

A total of 232,724,423 high-quality SNPs (allele frequency > 0.05 and call rate > 0.8) were identified in the samples. Heterozygosity rates ranged from 0.65% to 2.99%. Genomic distribution analysis revealed that 18.85% of SNPs were located in intergenic regions, 25.79% in intronic regions, and 31.94% in coding sequence (CDS) regions. The Cohen’s d values for *Z. loczyi* phenotypic variation among desert populations ranged from 0.2 to 0.5 (Table 2), indicating small but consistent differences in the traits among deserts. Meanwhile, the Q_st values of *Z. loczyi* populations consistently exceeded F_st values, suggesting that environmental selection plays a leading role in the phenotypic differentiation of *Z. loczyi* populations.

**Figure 6:**
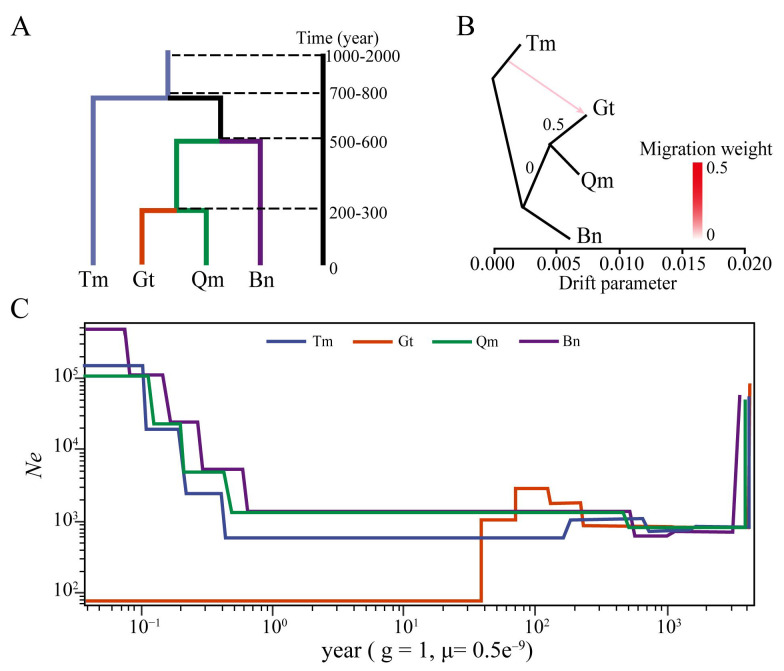
Differentiation of *Z. loczyi* population in four deserts. (**A**) divergence time of populations, (**B**) gene flow analysis of population, and (**C**) effective population size.

An analysis of SNP differentiation revealed a three-stage divergence pattern among the four desert populations of *Z. loczyi* (Figure 6A). Tm differentiated once in about 700–800 years, differentiated again in about 500–600 years to differentiate into Qm and Bn, and expanded in about 200–300 years to differentiate Gt from Bn (Figure 6A). The gene flow of *Z. loczy* was very low among the four deserts, with only a very weak gene flow between Tm and Gt (Figure 6B). Among the changes in the effective population size of *Z. loczy*, the effective population size of Gt is the smallest, and predictions suggest that their population sizes will eventually converge (Figure 6C). These results indicate that after the historical expansion of the *Z. loczyi*, the local adaptation of the population was affected neutrally due to limited gene flow, resulting in phenotypic differentiation. However, the population of Gt is under greater pressure to survive, resulting in a smaller effective population.

## 4. Discussion

### 4.1. Phenotypic Differentiation and Variation in Z. loczyi Populations

Significant differences in morphological traits—including plant height, flower size, and fruit size—were observed among populations from the four studied deserts, indicating high phenotypic diversity. Phenotypic variation indirectly reflects the extent of diversity within plant populations, with a higher coefficient of variation corresponding to greater community-level trait variability [15]. Phenotypic differentiation among populations of *Z. loczyi*, these findings align with prior research [42]. Notably, inter-population variation across different deserts exceeded intra-population variation within the same desert, suggesting local environmental adaptation. Population-level differences were the primary source of phenotypic variation, particularly in leaf and flower-related traits. This divergence may result from trade-offs in the phenotypic traits of *Z. loczyi* to optimize fitness in specific habitats. Plant phenotypic traits exhibit both conservatism and plasticity, influenced by genetic constraints and environmental factors [43]. In *Z. loczyi* populations, leaf and flower traits showed high plasticity, whereas fruit traits were more conservative, reflecting adaptive strategies to balance vegetative and reproductive growth under variable desert conditions. Despite population-specific differences, the continuous variation in traits underscores the species’ broad adaptability to diverse desert environments.

### 4.2. Response of Phenotypic Traits to Environmental Factors

Spatiotemporal environmental heterogeneity, including habitat variability and climate fluctuations, may promote genetic divergence at both specific and intraspecific levels, thereby influencing the biogeographic distribution of plant populations [44]. The four studied deserts exhibited significant differences in environmental conditions (e.g., precipitation, temperature, and soil properties), leading to divergent phenotypic traits among populations. To analyze environmental effects, we selected eight meteorological and six habitat variables via PCA, followed by RDA to identify key phenotypic traits responsive to environmental gradients. Temperature and precipitation are recognized as major drivers of plant functional trait variation [33,39]. Mixed-effects model predictions revealed that leaf and flower traits in the Gt population were most sensitive to water and heat changes, resulting in reduced leaf organ sizes. In contrast, the Qm population, occurring at higher altitudes with less water limitation, showed increased flower size under warmer conditions, potentially due to enhanced photosynthetic carbon assimilation. Proximity to rivers likely provided additional water resources, promoting phenotypic differentiation in local populations [29]. Phenotypic variation in plant populations reflects local climatic adaptation [45]. Our models indicated that the *Z. loczyi* populations of the four deserts improved their local adaptation by altering the traits of leaves and flowers in response to changes in environmental factors, with the Gt population exhibiting the strongest response to water and heat conditions.

### 4.3. Phenotypic Validation of SNP Genetic Differentiation

Phenotypic traits of *Z. loczyi* are continuous and minor variations in the four deserts, and phenotypic divergence was primarily driven by natural selection rather than genetic drift, reflecting local adaptation to heterogeneous desert environments. The population in the Tm underwent three major differentiation events from approximately 800 years ago to the present, subsequently differentiating into the Qm, Bn, and Gt populations, indicating that the distribution range of *Z. loczyi* was expanding, and this was consistent with previous events of the expansion of desert plants [46,47]. Notably, the Gt population experienced a contraction within the last 50 years. These populations of *Z. loczyi*, inhabiting distinct desert environments, exhibit phenotypic diversity shaped by genetic drift and environmental selection during differentiation. Investigating phenotypic variation and genetic differentiation along environmental gradients or across distinct habitat types indicates local adaptive evolution of plant species [48]. The near absence of gene flow among populations suggests geographic isolation as a primary driver of their genetic diversity [49,50,51]. This isolation creates spatial barriers that disrupt gene flow, profoundly affecting effective population size, genetic drift, and inbreeding degree, ultimately leading to inter-population genetic variation of *Z. loczyi*. Populations with larger effective population sizes exhibit greater capacity to overcome gene flow constraints, thereby enhancing their potential for local adaptation [52]. The desert-dwelling *Z. loczyi* exhibits both significant genetic differentiation and parallel adaptive convergence across extreme desert populations. Previous studies demonstrate that under conditions of restricted gene flow, the allelic effects associated with local adaptation follow an exponential distribution pattern [31]. Geographic isolation has resulted in the complete absence of gene flow among the four desert populations of *Z. loczyi*. The Gt population contraction resulted in reduced effective population size and accelerate drift, a stronger selective pressure influenced by hydrothermal conditions. Phenotypic diversity reflects substantial genetic and adaptive variation, enhancing populations’ capacity to adapt to local environmental changes [53,54]. This adaptive pattern is particularly evident in extreme desert environments. In conclusion, the rich phenotypic and genetic diversity in *Z. loczyi* populations enhances their local adaptation to extreme desert environments.

## 5. Conclusions

Through field investigations of 20 *Z. loczyi* populations across four deserts, we observed significant phenotypic variation among desert regions. The Tm population exhibited the smallest plant height and stem diameter, Gt populations have reduced leaf dimensions, while the Qm population showed the largest flower size. Inter-population phenotypic variation across different deserts exceeded intra-population variation within the same desert, demonstrating continuous clinal variation patterns. Leaf and flower traits were particularly sensitive to environmental factors. Mixed-effects models indicated that geographic location significantly influenced plant phenotypes, with temperature and precipitation being the primary drivers of leaf and flower trait variation. Notably, the Gt population displayed the strongest phenotypic responses to hydrothermal conditions. Compared with genetic drift, *Z. loczyi* is more affected by environmental selection and geographical isolation. This phenotypic variation reflects underlying genetic diversity shaped by geographic isolation and environmental heterogeneity—key factors enhancing local adaptation. While our study established correlations between phenotypic traits and molecular genetic diversity, the mechanistic links between phenotypic plasticity and genetic inheritance require further investigation. Future studies should integrate transcriptomic and epigenetic approaches to better elucidate *Z. loczyi* adaptive evolution in desert ecosystems. These findings advance our understanding of local adaptation mechanisms in extreme environments and provide foundational data for identifying stress-resistance genes in desert plants.

## Figures and Tables

**Table 1:** Phenotypic differentiation coefficients of *Z. loczyi* traits in four deserts.

Phenotypic Traits	Coefficient of Phenotypic Variation Among Populations (%)					
	Same Desert				Different Deserts	*p*-Value
	Tm	Gt	Qm	Bn		
Height	6.63	3.50	3.32	4.66	5.82	>0.05
Stem diameter	6.16	3.02	2.03	7.51	9.20	<0.05
Blade length	2.01	1.60	1.63	2.65	5.26	<0.001
Blade width	2.26	1.80	2.02	3.01	9.66	<0.001
Sepal length	4.99	1.71	1.89	2.05	7.52	<0.01
Sepal width	5.80	2.88	2.81	3.13	4.06	>0.05
Petal length	5.50	2.05	2.61	2.64	7.97	<0.01
Petal width	12.01	2.78	2.65	16.74	1.85	>0.05
Peduncle	7.18	2.58	2.29	13.67	9.80	>0.05
Stamen	4.36	2.04	2.15	2.79	10.24	<0.001
Fruit length	2.88	1.75	9.06	4.94	4.30	>0.05
Fruit width	2.96	2.98	4.95	2.56	4.82	>0.05

**Table 2:** Differences in population differentiation of *Z. loczyi*.

	Cohen’s d	Q_st	F_st
Tm_vs_Gt	0.34	0.38	0.06
Gt_vs_Qm	0.37	0.39	0.04
Qm_vs_Bn	0.50	0.37	0.02
Tm_vs_Qm	0.34	0.33	0.07
Gt_vs_Bn	0.19	0.39	0.04
Tm_vs_Bn	0.36	0.36	0.05

## Data Availability

The original contributions presented in this study are included in the article/Supplementary Materials. Further inquiries can be directed to the corresponding author.

## Supplementary Materials

The following supporting information can be downloaded at: https://www.mdpi.com/article/10.3390/genes16070729/s1. Table S1: Geographic information of *Z. loczyi* population collection; Table S2: Abbreviated comparison table of environmental factors; Table S3: List of all provenance climatic variables considered and their loadings on the principal component analysis of the four deserts; Table S4: List of all provenance Habitat variables considered and their loadings on the principal component analysis of the four deserts; Figure S1: Correlation analysis of phenotypic traits of *Z. loczyi*; Figure S2: Fixed effects predicted phenotypic traits of *Z. loczyi*.
