# Supplementary material for: Local Adaptation Shapes Phenotypic and Genetic Diversity in Zygophyllum loczyi
Source: Genes (Basel). 2025 Jun 23;16(7):729. doi: 10.3390/genes16070729 (PMC12294502; doi:10.3390/genes16070729)
Supplement: Supplementary file 1 [file genes-16-00729-s001.zip › genes-3677522-supplementary.pdf]

Local Adaptation Shapes Phenotypic and Genetic Diversity in *Zygophyllum loczyi*

Jan-Cheng Wang [1,2,3,*,†], De-Yan Wu [1,2,4,†], Xue-Rong Li [1,2], Jia-Yi Lu [1,2,5], Suo-Min Wang [6], Qing Ma [6], Hai-Shuang Liu [6], Xi-Yong Wang [1,2,3], Jing-Dian Liu [1,2,5] and Dao-Yuan Zhang [1,2,3]

1 State Key Laboratory of Desert and Oasis Ecology, Key Laboratory of Ecological Safety and Sustainable Development in Arid Lands, Xinjiang Institute of Ecology and Geography, Chinese Academy of Sciences, Urumqi 830011, China; wangxy@ms.xjb.ac.cn (X.-Y.W.); ariiiiiink@gmail.com (J.-D.L.);zhangdy@ms.xjb.ac.cn (D.-Y.Z.)
2 Xinjiang Key Laboratory of Conservation and Utilization of Plant Gene Resources, Xinjiang Institute of Ecology and Geography, Chinese Academy of Sciences, Urumqi 830011, China
3 Turpan Eremophytes Botanical Garden, Chinese Academy of Sciences, Turpan 838008, China
4 Institute of Economic Forest, Xinjiang Academy of Forestry, Urumqi 830092, China
5 College of Forestry and Landscape Architecture, Xinjiang Agricultural University, Urumqi 830052, China
6 State Key Laboratory of Herbage Improvement and Grassland Agro-Ecosystems, College of Pastoral Agriculture Science and Technology, Lanzhou University, Lanzhou 730020, China
* Correspondence: www-1256@ms.xjb.ac.cn
† These authors contributed equally to this work.

The following Supporting Information is available for this article:

Table S1 Geographic information of *Z. loczyi* population collection

Table S2 Abbreviated comparison table of environmental factors

Table S3 List of all provenance climatic variables considered and their loadings on the principal component analysis of the four deserts

Table S4 List of all provenance Habitat variables considered and their loadings on the principal component analysis of the four deserts

Figure S1 Correlation analysis of phenotypic traits of *Z. loczyi*

Figure S2 Fixed effects predicted phenotypic traits of *Z. loczyi*

Table S1 Geographic information of *Z. loczyi* population collection

Location	Pop	Latitude	Longitude
Tm	1	80.91245	41.43036
	2	77.35496	37.60674
	3	77.67286	37.79617
	4	78.25619	37.50903
Gt	1	88.79777	44.94489
	2	89.47241	44.77141
	3	89.97273	44.60738
	4	83.32866	44.57459
Qm	1	97.23306	37.12462
	2	95.60336	37.45898
	3	97.33404	37.1416
	4	95.37743	37.5725
	5	95.28757	37.88953
	6	91.03996	38.09801
Bn	1	100.5599	39.71043
	2	100.8024	39.58746
	3	98.79633	39.89502
	4	101.5154	39.19226
	5	103.1377	41.68567
	6	102.9259	38.4425

Table S2 Abbreviated comparison table of environmental factors

Type	Factor	Unit or	Describe
	Bio_1	℃	Annual Mean Temperature
	Bio_2	℃	Mean Diural Range
	Bio_3	ratio	Isothermality (Bio_2 /Bio_7) (×100)
	Bio_4	%	Temperature Seasonality (standard
	Bio_5	℃	Max Temperature of Warmest
	Bio_6	℃	Min Temperature of Coldest Month
	Bio_7	℃	Temperature Annual Range (Bio_5
	Bio_8	℃	Mean Temperature of Wettest
	Bio_9	℃	Mean Temperature of Dries'
	Bio_10	℃	Mean Temperature of Warmest
	Bio_11	℃	Mean Temperature of Coldest
	Bio_12	mm	Annual Precipitation
	Bio_13	mm	Precipitation of Wettest Month
	Bio_14	mm	Precipitation of Driest Month
	Bio_15	%	Precipitation Seasonality
	Bio_16	mm	Precipitation of Wettest Quarter
	Bio_17	mm	Precipitation of Driest Quarter
	Bio_18	mm	Precipitation of Warmest Quarter
	Bio_19	mm	Precipitation of Coldest Quarter
	Srad_01	$kJ\ m^{-2}\ d^{-1}$	Solar Radiation in January
	Srad_07	$kJ\ m^{-2}\ d^{-1}$	Solar Radiation in July
	Wind_01	$M \cdot s^{-1}$	Mean Wind Speed in January
	Wind_07	$M \cdot s^{-1}$	Mean Wind Speed in July
	alt	m	Altitude
	slope	°	Slope
	aspect	-	Aspect
	pH	-	pH
	Soil_type	-	Soil_type
	Sand	%	Sand to Soil Ratio
	Silt	%	Silt to Soil Ratio
	Clay	%	Clay to Soil Ratio
	Clcd	-	Land-use Type
	NDVI	-	Normalized Difference Vegetation
	River	-	Euclidean Distance from River

Table S3 List of all provenance climatic variables considered and their loadings on the principal component analysis of the four deserts

Provenance climatic variable	PC1	PC2	PC3	PC4
bio1	-3.15	0.97	-0.46	-0.33
bio10	-0.9	0.47	-0.25	-0.07
bio11	-2.94	-0.39	0.11	0.21
bio12	2.07	-0.62	0.08	-0.13
bio13	-1.05	-0.35	0.57	-0.17
bio14	-6.42	-0.85	-0.51	-0.16
bio15	1.46	0.35	0.67	-0.02
bio16	0.84	-0.48	0.51	-0.16
bio17	-3.47	-0.65	-0.4	0.04
bio18	0.81	-0.45	0.55	-0.16
bio19	-3.07	-0.54	-0.35	0.24
bio2	-1.63	0.45	0.2	0.08
bio3	-0.14	0.45	0.31	0.12
bio4	7.16	-0.06	-0.51	0.02
bio5	-0.21	0.42	-0.2	-0.03
bio6	-1.31	0.02	0.1	0.16
bio7	0.72	0.23	-0.14	0.05
bio8	-0.94	0.5	-0.22	-0.06
bio9	-3.42	-0.23	0.01	0.04
sr1	11.04	0	-0.11	0.09
sr2	10.37	-0.21	-0.23	-0.03
wid1	-3.66	0.5	0.18	0.12
wid2	-2.15	0.45	0.11	0.13

Table S4 List of all provenance Habitat variables considered and their loadings on the principal component analysis of the four deserts

Provenance habitat variable	PC1	PC2	PC3	PC4
altitude	-6.39	-1.63	0	0.01
aspect	-1.56	0.19	-0.28	0.44
clay	3.73	-0.19	0.46	0.17
pH	4	0.33	0.04	-0.08
latitude	-1.14	-0.03	0.02	-0.04
longitude	0.48	0.19	-0.03	-0.19
NDVI	-0.12	0.03	-0.03	-0.14
clcd	-0.95	0.02	-0.04	-0.18
river	-12.94	0.88	0	-0.02
silt	1.92	0.07	0.22	-0.02
slope	7.41	-0.22	-0.43	-0.06
soil-type	5.55	0.38	0.08	0.1

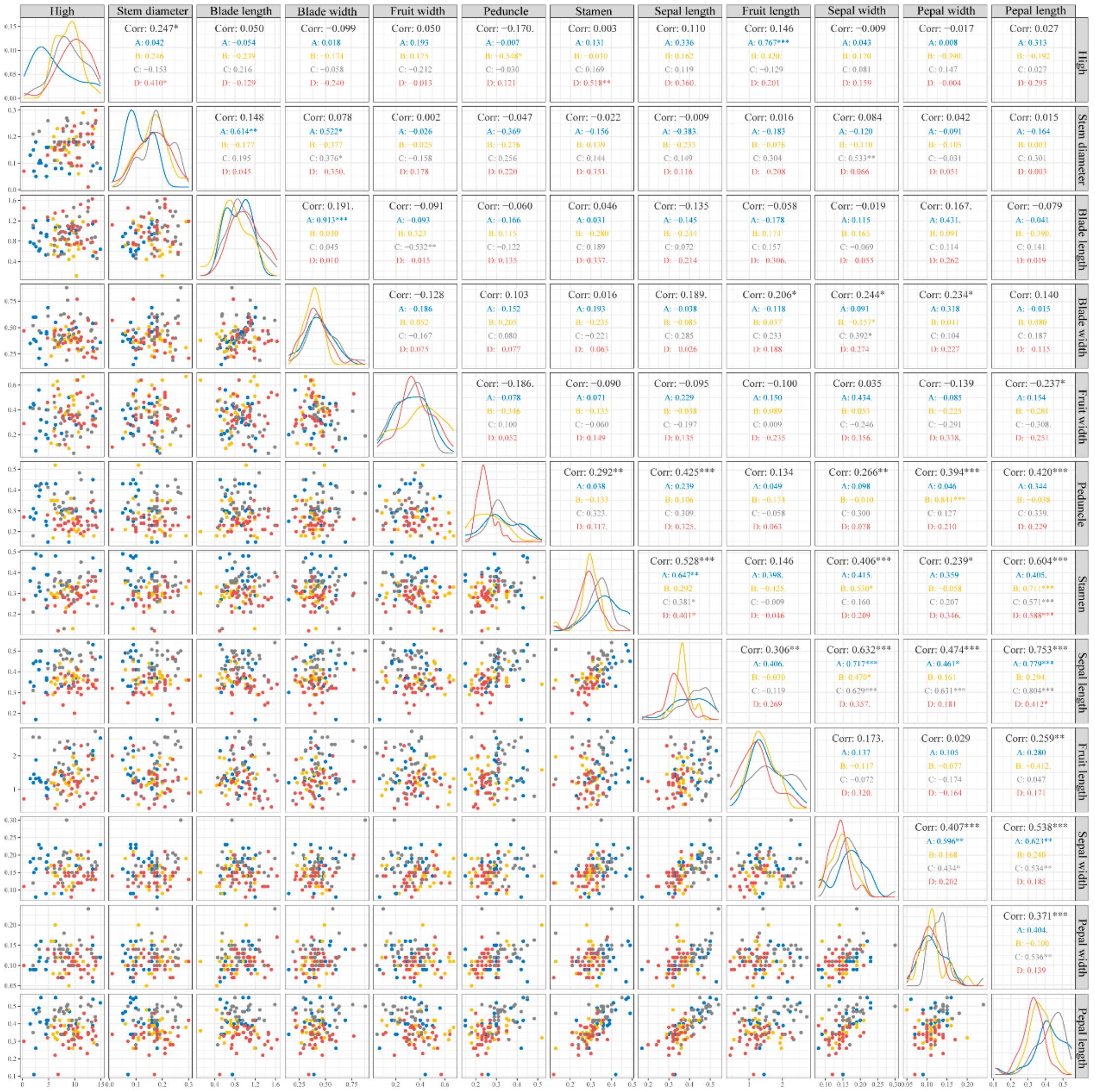

Figure S1 Correlation analysis of phenotypic traits of *Z. loczyi*

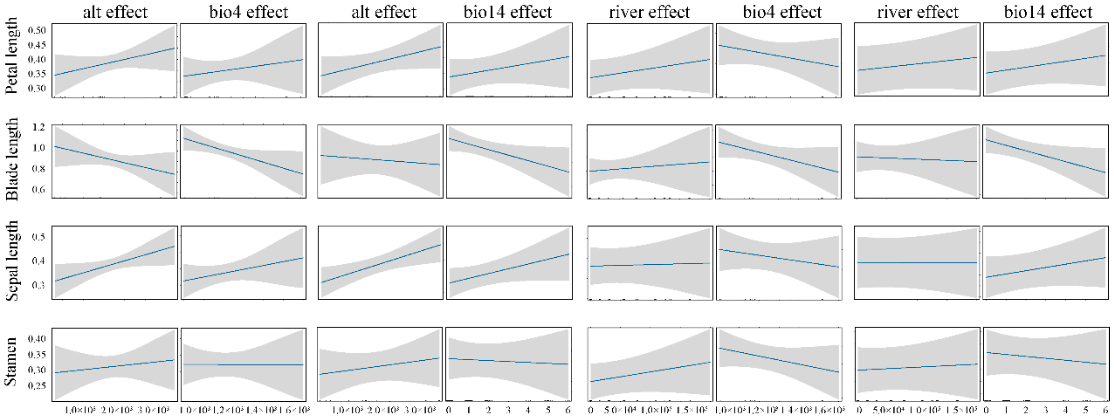

Figure S2 Fixed effects predicted phenotypic traits of *Z. loczyi*
